# Engagement in a program promoting lifestyle modification is associated with better patient-reported outcomes for people with MS

**DOI:** 10.1007/s10072-015-2089-1

**Published:** 2015-02-01

**Authors:** Emily J. Hadgkiss, George A. Jelinek, Keryn L. Taylor, Claudia H. Marck, Dania M. van der Meer, Naresh G. Pereira, Tracey J. Weiland

**Affiliations:** 1 Emergency Practice Innovation Centre, St Vincent’s Hospital Melbourne, PO Box 2900, Fitzroy, VIC 3065 Australia; 2 Department of Epidemiology and Preventive Medicine, Monash University, Melbourne, VIC Australia; 3 Department of Psychiatry, St Vincent’s Hospital Melbourne, PO Box 2900, Fitzroy, VIC 3065 Australia; 4 Department of Medicine, St Vincent’s Hospital, The University of Melbourne, Melbourne, VIC Australia; 5 Faculty of Medicine, Notre Dame University, PO 1225, Fremantle, WA 6959 Australia

**Keywords:** Multiple sclerosis, Quality of life, Engagement, Lifestyle modification, Prevention

## Abstract

There is increasing interest in patient-centered approaches to chronic disease management and prevention. For people with multiple sclerosis (PwMS), patient empowerment plays a role in improving a range of health-related outcomes. This study aimed to compare health-related quality of life (HRQOL), fatigue, and depression risk between people who have and have not attended a week-long physician-led residential educational retreat or accessed other self-help resources (a book and online content) that foster patient empowerment including the adoption of healthy lifestyle behaviors. PwMS were recruited to the study using online platforms and asked to complete a comprehensive online survey. Data from 2,233 respondents were analysed. Bivariate results indicated that PwMS who had attended a retreat (*n* = 247), read the associated book (*n* = 1,167) or regularly visited online sites promoting lifestyle modification (*n* = 795), had better HRQOL and lower rates of depression and fatigue than those who had not. The depression risk among retreat attendees (8.6 %) was around half that of the whole sample. Regression analysis showed that, controlling for age and gender, compared to the highest level of engagement, no engagement with the resources was associated with nearly threefold higher odds of clinically significant fatigue, tenfold higher odds of depression risk, and physical and mental HRQOL scores 19.5 and 15.6 points lower, respectively. These results are congruent with previously reported post-retreat improvements in HRQOL, and strongly support a role for patient engagement in resources promoting lifestyle modification. Physicians should encourage more active involvement of PwMS in their own health care.

**Electronic supplementary material:**

The online version of this article (doi:10.1007/s10072-015-2089-1) contains supplementary material, which is available to authorized users.

## Introduction

Multiple sclerosis (MS) is a chronic inflammatory disorder of the central nervous system often diagnosed in young adults. In the absence of a known cure, a focus of disease management is on maintaining function and quality of life (QOL). There is growing evidence that lifestyle factors may improve quality of life, reduce relapse rate and slow the progression of the disease [1–6]. Modifying lifestyle and health behaviors requires commitment but the potential benefits in MS, as well as a reduced risk of other chronic lifestyle-related diseases, are significant.

There has been a paradigm shift in the management of chronic diseases towards a patient-centered approach to self-management and prevention. People who are proactive in their health may achieve better outcomes than those more passive. Self-efficacy and patient activation have positive associations with healthy lifestyle behaviors, better QOL and functional status, fewer health visits, and decreased depressive symptoms [7–9]. For people with MS (PwMS), increasing self-efficacy predicts improvements in walking ability and physical and psychological impact of MS [10], lower depression scores and better quality of life [11].

A physician-led patient-centered risk modification program, the Overcoming Multiple Sclerosis (OMS) Program, aims to educate PwMS about secondary and tertiary prevention and provide greater autonomy about health care decisions [12, 13]. The 5-day live-in retreat focuses on modifiable lifestyle factors that can be addressed by an individual, ideally with the support of a health professional, empowering participants to take control of their own health. The program content was developed in 1999 from an appraisal of peer-reviewed medical literature on the role of lifestyle factors in MS progression, and is continuously updated to integrate the most recent evidence [3]. The retreat, delivered by medical practitioners with support from trained counsellors, offers an intensive, experiential week, where participants talk about experiences, ask questions, eat the recommended diet, exercise and meditate while forming new friendships.

The OMS program is supported by resources, including a book [3], a website [14] and social media sites including Facebook and Twitter. Each delivers similar content, with differences. The book is detailed with chapter summaries and tables to guide a lay person through the complex information. The website reviews similar content but, along with the social media, is regularly updated with new research. The online platforms encourage participation, contribution to a forum, commentary, sharing of recipes or inspiring stories. The website, established in 2008, has approximately 2,800 visitors per day, two-thirds first time visitors, mostly from North America, with over 11,000 members of the forum.

A longitudinal analysis of the retreats showed clinically and statistically significant improvements in health-related quality of life (HRQOL) compared to baseline (prior to retreat attendance); however, interpretation was limited by the absence of a control group [12, 13]. The Health Outcomes and Lifestyle Intervention in a Sample of People with Multiple Sclerosis study (the HOLISM study [2, 4, 6, 15]) enrolled PwMS from 57 countries via social media platforms, examining the association of lifestyle risk factors with disease outcomes. This cohort of approximately 2,500 people with different types of MS comprises a substantial subset who have previously attended an OMS retreat or accessed other OMS resources, including many not participating in the longitudinal analysis because they attended an OMS retreat in a different location (Coromandel, New Zealand or Perth, Australia) than Victoria, Australia.

This unique dataset provides an opportunity to compare health outcomes of those attending an OMS retreat with those not, and between people engaged in other retreat-linked OMS resources, and those not. The aim of this study was to examine differences in patient-reported outcomes (HRQOL, depression, fatigue) between people attending an OMS retreat and those not. A secondary aim was to explore associations between these outcome measures and engagement with the other retreat-linked OMS resources (book or online content).

## Materials and methods

### Methods and tools

The methodology of the HOLISM study has been described in detail [15]. Using Web 2.0 platforms (social media, interactive websites, blogs, forums), participants over 18 years of age with a medical diagnosis of MS were recruited and completed an online questionnaire. Contact details were recorded for follow-up. Data were stored in a password-protected, de-identified database. Ethical approval was granted by St Vincent’s Hospital Melbourne Ethics Committee (LRR 055/12).

The independent variables pertained to participants’ level of engagement with OMS resources. Three items asked if they had attended a live-in retreat, if they had read the book, and how frequently they had visited the website or corresponding social media pages (Facebook and Twitter).

Outcome measures were Multiple Sclerosis Quality of Life (MSQOL-54) [16] for HRQOL, the Fatigue Severity Scale (FSS) [17] for fatigue, with a mean score ≥4 indicating clinically significant fatigue, and the Patient Health Questionnaire depression module short version (PHQ-2) with a cut off score ≥3 [18] to screen for depression.

### Statistical analyses

Data were analysed using IBM SPSS Statistics 21.0. Continuous data were summarized using mean, 95 % confidence interval (CI) and categorical data using number and percentage. Bivariate analysis explored the association between each ‘engagement’ item (retreat, book and website) and HRQOL, depression and fatigue. Independent samples *t* test compared two groups on continuous outcome measures. Categorical data involving two by two contingency tables were analysed with Fisher’s exact test. Adjusted standardized residuals indicated under- or over-representation of groups with a cutoff set at ±2.0.

For regression analysis, items on retreat, book, and website use were combined into one variable with eight levels. Two levels within the engagement category containing five or fewer cases were excluded so as not to give rise to unreliable estimates. The remaining six groups within the ‘engagement’ variable were compared using those with no exposure to any of the three resources as the reference category. Multiple regression (enter method) was used to determine whether levels of engagement were significant predictors of HRQOL, controlling for age and gender. Binary logistic regression (enter method) was used to determine the adjusted odds ratio of screening positive for depression or clinically significant fatigue (controlling for age and gender). Two-tailed tests of significance were used with significance set at 0.05.

## Results

Up to 2,233 (90.4 %) of the sample of people with confirmed MS (*n* = 2,469) completed items on engagement. Overall, 11.1 % of respondents had participated in an OMS retreat, 52.3 % had read the OMS book, and 35.6 % accessed the OMS website regularly (Table 1). A total of 559 participants (25.0 %) had never accessed any of the resources.

**Table 1:**
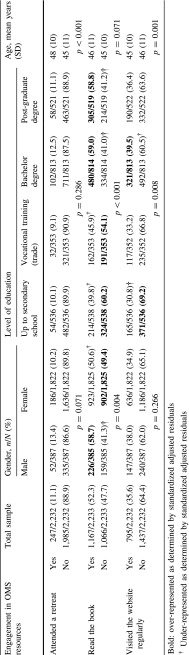
Summary of engagement responses by demographics

### Demographic associations

Retreat attendees were older on average (48 vs. 45 years, *p* < 0.001) but there was no significant difference for gender or level of education. Males were more likely to have read the book (58.7 vs. 50.6 %, *p* = 0.004), and those with a bachelor or postgraduate degree (*p* < 0.001), but there was no association with age. Website users were slightly younger (45 vs. 46 years, *p* = 0.001) and more likely to have a bachelor degree (*p* = 0.008) but there was no significant difference for gender (Table 1).

### Health-related quality of life

Retreat attendees had higher mean physical health composite (PHC) and mental health composite (MHC) scores than non-attendees (*p* < 0.001), with the highest mean PHC (66.6) and MHC (75.4) scores; significant differences were also observed between those who had read the book or regularly accessed the website, compared to those who had not. The mean score difference (compared to those not engaged in the activity) was most pronounced for the PHC and MHC scores of those who had read the book (Table 2).

**Table 2:** Physical and mental health composite scores by engagement with Overcoming Multiple Sclerosis resources

Engagement in OMS resources		Physical health composite				Mental health composite			
		*n*	Mean score	95 % CI	*p*	*n*	Mean score	95 % CI	*p*
Attended a retreat	Yes	207	66.6	64.0–69.2	**<0.001**	235	75.4	73.1–77.6	**<0.001**
	No	1,668	58.5	57.4–59.5		1,903	66.1	65.1–67.1	
Read the book	Yes	974	65.6	64.3–66.9	**<0.001**	1,117	71.8	70.7–73.0	**<0.001**
	No	899	52.6	51.2–54.0		1,020	61.9	60.6–63.3	
Visited the website regularly	Yes	676	63.8	62.2–65.4	**<0.001**	768	69.2	67.7–70.7	**0.001**
	No	1,197	56.9	55.7–58.1		1,369	66.1	64.9–67.2	

After controlling for gender (not a significant covariate) and age, those attending a retreat as well as reading the book and visiting the website regularly had PHC scores 19.5 points higher than those with the lowest level of engagement (‘none’) (Table 3). This was followed by the book and website group, the book and retreat group and the book only group. Website only users were not significantly different from those not engaging in any resources. Similar results were observed in the MHC scores; those with the greatest engagement (all three resources) had scores 15.6 points higher than no engagement (Table 3). Participants accessing the website only had lower scores than those not engaging with any resources. The models explained 14.5 % of variance for the PHC and 6.3 % of variance for the MHC.

**Table 3:** Predictors of physical and mental health composite scores

	Covariate	Grouping	*B*	95 % confidence interval	*p*
Physical Health Composite	Age		−0.5	−0.5 to −0.4	**<0.001**
	Gender	Male	1.3	−1.1 to 3.7	0.276
	Engagement with OMS resources	All three	19.5	15.4 to 23.5	**<0.001**
		Book and retreat	11.0	6.6 to 15.4	**<0.001**
		Book and website	13.2	10.7 to 15.6	**<0.001**
		Book only	10.3	7.8 to 12.9	**<0.001**
		Website only	−3.3	−6.7 to 0.1	0.059
		None (reference)	–	–	–
Mental Health Composite	Age		0.1	0.0 to 0.2	**0.048**
	Gender	Male	−0.4	−2.8 to 1.9	0.735
	Engagement with OMS resources	All three	15.6	11.7 to 19.6	**<0.001**
		Book and retreat	8.3	4.1 to 12.6	**<0.001**
		Book and website	8.3	5.9 to 10.7	**<0.001**
		Book only	7.0	4.6 - 9.4	**<0.001**
		Website only	−5.0	−8.3 to −1.8	**0.002**
		None (reference)	–	–	–

Adjusted R Squared: PHC, 0.145; MHC, 0.063

Bold indicate statistical significance

*B* Unstandardized regression coefficient

HRQOL composite scores between participants who had and had not attended a retreat were compared with results reported in a previous study by the same research group [12], showing pre- and post-retreat (one year) attendance (Table 4). The two studies shared strong similarities in HRQOL outcomes.

**Table 4:** Comparison of median health-related quality of life scores across two studies of HRQOL after OMS retreat attendance

	Study 1: current study					Study 2: previous study				
	Retreat non-attendee		Retreat attendee		Median difference (%)	Baseline (prior to retreat attendance)		Post-one year retreat attendance		Median difference (%)
	*n*	Median (IQR)	*n*	Median (IQR)		*n*	Median (IQR)	*n*	Median (IQR)	
Physical Health Composite	1,668	57.8 (41.7–76.9)	207	68.2 (53.0–83.4)	10.4 (18.0)	190	63.7 (48.6–77.8)	190	75.5 (56.4–84.9)	11.8 (18.5)
Mental Health Composite	1,903	71.3 (50.3–83.6)	235	80.6 (66.0–88.5)	9.3 (13.0)	178	72.9 (51.9–85.1)	178	81.5 (68.7–90.3)	8.6 (11.8)

Study 1: HOLISM study

Study 2: Longitudinal study of the effect of a residential retreat [17]

Scores shown here are median (interquartile range), for the purpose of comparison

### Fatigue

Participants attending a retreat were significantly less likely to screen positive for fatigue. Results were similar for respondents who had read the book or accessed the website regularly. The greatest difference observed was among those who had read the book compared to those who had not (see supplemental material).

With the exception of regular ‘website only’ users, all groups had reduced odds of fatigue compared to those not engaged, even after controlling for gender (not significant) and age (OR 1.024, 95 % CI 1.014–1.033, *p* < 0.001) (Table 5). The greatest reduction in odds was for those engaged in all three resources (OR 0.357, 95 % CI 0.239–0.535, *p* < 0.001).

**Table 5:** Predictors of fatigue and depression risk

Covariate	Grouping	FSS mean score ≥4			PHQ-2 score ≥3		
		Exp (B)	95 % confidence interval	*p*	Exp (B)	95 % confidence interval	*p*
Age		1.024	1.014–1.033	**<0.001**	0.993	0.982–1.003	0.173
Gender	Male	0.798	0.626–1.018	0.069	1.089	0.812–1.460	0.570
Engagement with OMS resources	All three	0.357	0.239–0.535	**<0.001**	0.102	0.037–0.281	**<0.001**
	Book and retreat	0.492	0.318–0.760	**0.001**	0.468	0.260–0.842	**0.011**
	Book and website	0.434	0.337–0.560	**<0.001**	0.410	0.296–0.569	**<0.001**
	Book only	0.422	0.328–0.544	**<0.001**	0.504	0.370–0.686	**<0.001**
	Website only	1.081	0.744–1.569	0.683	1.372	0.977–1.927	0.068
	None (reference)	–	–	–	–	–	–

Exp (B): Adjusted odds ratio

Pseudo R Squared (Nagelkerke): fatigue = 0.073; depression = 0.067

Bold indicate statistical significance

### Depression

Similarly, those attending a retreat had the lowest proportion screening positive for depression (8.6 %). Those who had read the book or visited the website regularly were also significantly less likely to screen positive (see supplemental material).

Neither age nor gender was a significant covariate. Engaging in OMS resources was associated with significantly reduced odds of screening positive for depression for all groups except those only visiting the website regularly (Table 5). The result was most pronounced for those engaging in all three resources, whose odds of a positive depression screen was reduced by 89.8 % (OR 0.102, 95 % CI 0.037–0.281, *p* < 0.001).

## Discussion

We have previously reported improved HRQOL in PwMS attending a residential retreat in Victoria, Australia, at 1, 2.5 and 5 years post-retreat compared with baseline [12, 13]. The large international cohort of PwMS in the HOLISM study comprises a significant proportion of people who have attended one of these retreats, and others of the same design elsewhere in Australia and in New Zealand. This allows a different method of assessment of the value of such retreats through comparison of HRQOL outcomes for those attending a retreat against others not attending. PwMS attending an OMS retreat have significantly better HRQOL than those who not. Retreat attendees also have markedly less fatigue or depression. The prevalence of the latter among this subgroup was around half that of the whole sample.

The findings are comparable in magnitude to our previous longitudinal cohort study where one year after a retreat, participants reported improvements in physical HRQOL of 11.8 points (18.5 %) and mental HRQOL of 8.6 points (11.8 %) [12]. These differences are similar to the median differences observed in the current study; those attending a retreat scored 10.4 points higher (18.0 %) on physical HRQOL and 9.3 points higher (13.0 %) on mental HRQOL. The congruence between these findings strengthens the previously reported results, suggesting that this specific retreat promoting patient empowerment and lifestyle change does significantly improve QOL. The HOLISM study also allowed us the opportunity to examine any benefit of retreat-linked resources. Those reading the OMS book also had significantly better HRQOL and lower prevalence of clinically significant fatigue than those not. Similar results were observed for participants who regularly accessed the OMS website, however, the differences with the comparison group, although statistically significant, were not as pronounced.

In regression analysis, participants who engaged in all three resources consistently observed better HRQOL, and markedly less fatigue or depression. The reference group, those with no engagement with any of the OMS resources, had around a tenfold and nearly threefold higher odds of positive screen for depression and clinically significant fatigue, respectively.

There were significantly better outcomes as participants’ engagement with resources increased, reflecting a cumulative effect of degree of engagement with the resources. The suite of OMS resources aims to educate PwMS about their condition and encourages them to consider the strength of evidence available on secondary and tertiary prevention through lifestyle modification. It appears that the adoption or maintenance of certain health behaviors may affect HRQOL, depression and fatigue in a complex way. Others have shown significant reductions in depression following participation in a lifestyle modification program [19].

Rather than the effect of lifestyle modification itself, or perhaps additional to that effect, perceived benefits for HRQOL, depression and fatigue may arise from participants’ level of empowerment or self-efficacy. Empowerment is both a process and an outcome—“a process to increase one’s ability to think critically and act autonomously… an outcome when an enhanced sense of self-efficacy occurs as a result of the process [20]”. This is distinguished from a patient simply becoming more compliant. This relates closely to our study; accessing the OMS resources may provide people with a greater internal ‘locus of control’ which may represent the sense of empowerment that proactive patients experience, giving rise to better outcomes. Additionally, actively engaged patients may be more likely to have a greater understanding of their condition, a better relationship with their health provider, be more likely to attend clinic appointments and adhere to their treatment regime, thus giving rise to better outcomes.

A further benefit from active engagement with OMS resources may be the social and emotional support that participants receive by interacting with other PwMS. Social support is a significant predictor of HRQOL [21] and having a greater number of positive experiences, including those that promote physical and mental health or involve social interaction, is associated with better QOL and fewer depressive symptoms [22]. Further, the OMS program encourages PwMS to have a positive outlook; having greater dispositional hope is thought to improve one’s ability to adjust to or cope with MS [23].

### Limitations

Data were self-reported; hence it is not possible to verify diagnosis of MS. However, a large online registry found 98.7 % accuracy for self-reported diagnoses of MS [24]. The sample is self selecting; participants volunteered to complete the questionnaire in their own time, without incentives. A self-selecting online sample may have a greater level of education or higher socioeconomic status than the general population. They, therefore, may not be representative of all PwMS. Further, given the prevalence of cognitive impairment in PwMS (particularly in secondary progressive MS), the representativeness of our sample may be limited by the inability of some people with cognitive impairment to participate. Additionally, as our participants were from a wide range of countries, difficulty in web access may have limited representativeness of the sample. The resources assessed in this study are not exclusive. There are many other books, websites and programs that participants may have accessed that also explore lifestyle modification. PwMS also have the option of accessing physician-led prevention programs in rehabilitation services if unable or unwilling to undertake such self-directed lifestyle modification.

Reverse causality should be considered as a possible contributor to the associations observed as people who have lower levels of depression, fatigue and higher HRQOL may be more likely to engage in educational resources. Lack of randomization precludes more definitive conclusions from our study, and the earlier study [12] to which we compared our data had only a small number of participants. Further, it is not possible to precisely evaluate the impact of the book or information provided by websites.

## Conclusion

People with MS attending residential retreats and actively engaged in resources that promote lifestyle modification have better mental and physical HRQOL, and a markedly lower prevalence of clinically significant fatigue and depression. Physicians should support and encourage PwMS to play a more active role in their health and to adopt healthy lifestyle behaviors. Intervention studies that seek to increase patient self-efficacy and promote lifestyle modification are urgently required.

## Electronic supplementary material

Below is the link to the electronic supplementary material.

Supplementary material 1 (DOCX 12 kb)
