# Supplementary material for: Engagement in a program promoting lifestyle modification is associated with better patient-reported outcomes for people with MS
Source: Neurol Sci. 2015 Feb 1;36(6):845–52. doi: 10.1007/s10072-015-2089-1 (PMC4454831; doi:10.1007/s10072-015-2089-1)
Supplement: Supplementary file 1 — Supplementary material 1 (DOCX 12 kb) [file 10072_2015_2089_MOESM1_ESM.docx]

Supplementary material: Fatigue and depression screen outcome by engagement with Overcoming Multiple Sclerosis resources

|  | | Fatigue risk | |  | Depression risk | |  |
| --- | --- | --- | --- | --- | --- | --- | --- |
| Engagement with resources | | Not clinically significant,  n(%) | Clinically significant,  n(%) | p | Negative depression screen,  n(%) | Positive depression screen,  n(%) | p |
| Attended a retreat | Yes | **103/237 (43.5)*** | 134/237 (56.5)† | **0.002** | **223/244 (91.4)*** | 21/244 (8.6)† | **<0.001** |
|  | No | 628/1887 (33.3)† | **1259/1887 (66.7)*** |  | 1562/1966 (79.5)† | **404/1966 (20.5)*** |  |
| Read the book | Yes | **486/1113 (43.7)*** | 627/1113 (56.3)† | **<0.001** | **1015/1155 (87.9)*** | 140/1155 (12.1)† | **<0.001** |
|  | No | 246/1011 (24.3)† | **765/1011 (75.7)*** |  | 771/1055 (73.1)† | **284/1055 (26.9)*** |  |
| Visited the website regularly | Yes | **300/757 (39.6)*** | 457/757 (60.4)† | **<0.001** | **668/793 (84.2)*** | 125/793 (15.8)† | **0.003** |
|  | No | 431/1367 (31.5)† | **936/1367 (68.5)*** |  | 1120/1417 (79.0)† | **297/1417 (21.0)*** |  |
| *(Bold) Over-represented as determined by standardized adjusted residuals  † Under-represented as determined by standardized adjusted residuals | | | | | | | |
